# Whole Exome Sequencing Identifies a Troponin T Mutation Hot Spot in Familial Dilated Cardiomyopathy

**DOI:** 10.1371/journal.pone.0078104

**Published:** 2013-10-29

**Authors:** Nzali Campbell, Gianfranco Sinagra, Kenneth L. Jones, Dobromir Slavov, Katherine Gowan, Marco Merlo, Elisa Carniel, Pamela R. Fain, Pierluigi Aragona, Andrea Di Lenarda, Luisa Mestroni, Matthew R. G. Taylor

**Affiliations:** 1 Cardiovascular Institute and Adult Medical Genetics, University of Colorado, Aurora, Colorado, United States of America; 2 Cardiovascular Department, “Ospedali Riuniti” and University of Trieste, Trieste, Italy; 3 Bioinformatics Shared Resource of the University of Colorado Cancer Center and the Department of Biochemistry and Molecular Genetics, University of Colorado School of Medicine, Aurora, Colorado, United States of America; 4 Human Medical Genetics and Genomics Program, University of Colorado School of Medicine, Aurora, Colorado, United States of America; 5 Cardiology Unit, Ospedale Trebisacce, Cosenza, Italy; Universite de Montreal, Canada

## Abstract

Dilated cardiomyopathy (DCM) commonly causes heart failure and shows extensive genetic heterogeneity that may be amenable to newly developed next-generation DNA sequencing of the exome. In this study we report the successful use of exome sequencing to identify a pathogenic variant in the *TNNT2* gene using segregation analysis in a large DCM family. Exome sequencing was performed on three distant relatives from a large family with a clear DCM phenotype. Missense, nonsense, and splice variants were analyzed for segregation among the three affected family members and confirmed in other relatives by direct sequencing. A c.517T C>T, Arg173Trp *TNNT2* variant segregated with all affected family members and was also detected in one additional DCM family in our registry. The inclusion of segregation analysis using distant family members markedly improved the bioinformatics filtering process by removing from consideration variants that were not shared by all affected subjects. Haplotype analysis confirmed that the variant found in both DCM families was located on two distinct haplotypes, supporting the notion of independent mutational events in each family. In conclusion, an exome sequencing strategy that includes segregation analysis using distant affected relatives within a family represents a viable diagnostic strategy in a genetically heterogeneous disease like DCM.

## Introduction

Dilated cardiomyopathy (DCM: OMIM 115200) is a disease characterized by progressive left ventricular dilation and systolic dysfunction affecting at least 1 in 2500 individuals [1] and is a major cause of heart failure and need for cardiac transplantation. In at least a third of cases there is evidence of a genetic etiology [2]–[5] and mutations in over thirty DCM genes have been described as leading to DCM in familial as well as sporadic cases. [3], [6] This genetic heterogeneity represents a diagnostic challenge and has led to the development of progressively larger and more costly DCM genetic testing panels for clinical diagnosis. However, even the largest of these gene-testing panels are only able to test for ‘known’ genes. Further, as new cardiomyopathy genes are identified the clinician must consider if a previously tested patient must be re-contacted and re-tested with the ‘updated’ panel. The entry of next-generation (NextGen) DNA sequencing into the clinic is transforming molecular diagnostics after several groups demonstrated successes in identifying pathogenic mutations in rare diseases.[7]–[13] In the cardiovascular arena, more prevalent genetic conditions including cardiomyopathies and channelopathies are currently evaluated using targeted multi-gene panels which have diagnostic sensitivities >40%, >65%, and >75% in the cases of DCM, hypertrophic cardiomyopathy, and long QT syndrome respectively.

The diagnostic limitations of current gene-panel approaches are potentially solvable by NextGen sequencing methods including whole exome sequencing, which has recently become clinically available and interrogates all annotated human genes. In theory, exome sequencing will identify mutations of the coding portions of all known cardiomyopathy genes as well as offering the potential to detect mutations in genes not yet associated with the phenotype. A recognized limitation of exome sequencing is that enormous large datasets are generated and that single individuals may harbor dozen of rare variants that make identifying a singular causative variant a difficult proposition. Leveraging exome data from two or more related and affected individuals within a family may address this challenge, particularly if they are distantly related. Norton et al. performed an elegant NextGen exome sequencing study using this strategy in a large family with familial dilated cardiomyopathy (DCM), where copy-number analysis ultimately identified an 8.7 kb intragenic deletion in *BAG3* causing the disease. [14] Other investigators have used whole-exome approaches to identify novel mutations in cases of rare autosomal recessive forms of hypertrophic cardiomyopathy or DCM, predominantly in pediatric cases.[15]–[18] In this study, we report the use of exome sequencing to identify a pathogenic nucleotide variant in a multigenerational, adult-onset family with DCM. Bioinformatic filtering of detected variants along with testing for shared variants among distantly affected relatives was used to narrow-down the list of possible causative variants and to ultimately identify a single *TNNT2* rare variant that segregated with the DCM phenotype in all affected relatives.

## Methods

### Subjects

**Figure 1:**
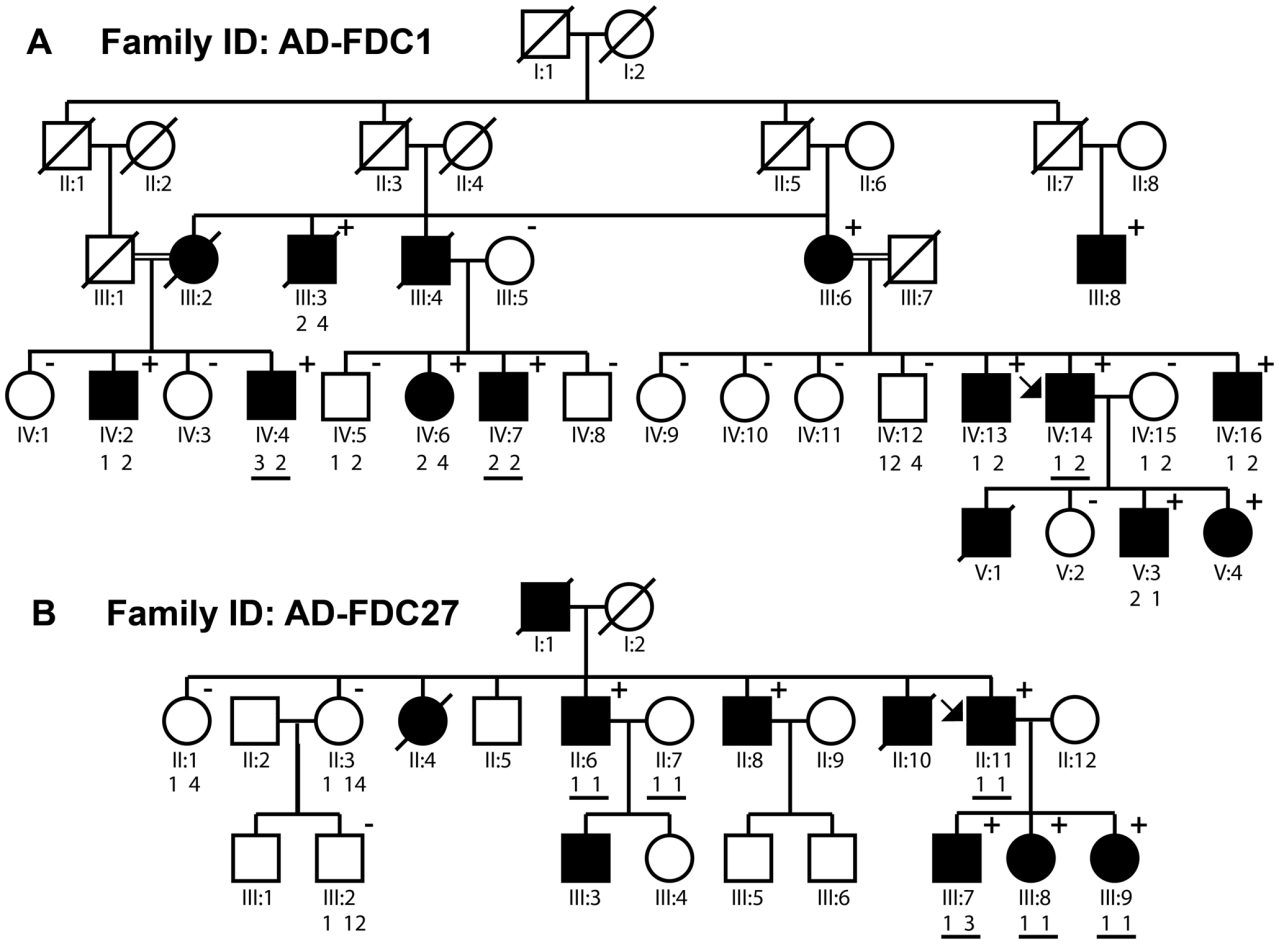
Pedigrees of families (A) AD-FDC1 and (B) AD-FDC27. The family structures of both TNNT2 mutation families are shown. The proband is indicated by an arrow. Males and females are depicted as squares and circles, respectively. Affected individuals are identified by shading. Presence or absence of the Arg173Trp variant confirmed by Sanger sequencing is indicated by plus (‘+’) and minus (‘−’) signs, respectively. Paired numbers beneath individuals represent the numbered haplotypes according to (Table 4); double-numbers represent recombinant haplotypes further detailed in the Table S3. Individuals studied by exome sequencing are indicated by present of black underline beneath haplotype numbering.

Subjects were enrolled through the Familial Cardiomyopathy Registry, a multicenter genetic study primarily focused in the United States and Italy. Detailed clinical information was obtained for each subject and included family history, age of presentation, initial symptoms of heart failure, New York Heart Association (NYHA) classification, physical examination, electrocardiograms, echocardiograms, and when appropriate Holter monitoring, exercise testing, invasive examination (right and left heart catheterization, ventriculography, coronary angiogram and endomyocardial biopsy). The diagnostic criteria for familial DCM followed the guidelines for the study of familial dilated cardiomyopathies based on major and minor criteria. [19] The major criteria were: a) left ventricular ejection fraction less than 45% or fractional shortening less than 25%, and b) left ventricular end-diastolic dimension >117% of the predicted value corrected for age and body surface area. [20] Individuals were classified as healthy when found to be normal or affected by known diseases, and unknown when isolated minor cardiac or skeletal muscle abnormalities were observed, as previously described. The largest family with familial DCM in the registry, AD-FDC1, was selected for initial study by exome sequencing based on the large number of affected and distantly related relatives (Figure 1). Written informed consent was obtained from all subjects and the “Ospedali Riuniti” and University Ethics Committee, Italy, and the Colorado Multiple Institutional Review Board (COMIRB, Protocol 99–177) Colorado, USA specifically approved this study. Prior to 1998, in Italian study subjects, written consent was not obtained, while oral informed consent was obtained according to the contemporaneous consent guidelines. Investigators provided informed consent by explaining the purpose of this study. Study subjects accepted to participate in the study and undergo clinical examination and DNA testing, as documented by their files, and the Italian and USA ethic committees approved this consent process.

### Exome Sequencing

Genomic DNA was extracted from whole blood in standard fashion. DNA (3–5 micrograms) was sheared, size selected (∼400–600 bp), ligated to sequencing adapters, and PCR amplified to enrich for targets to sequence following the standard Illumina TruSeq library preparation (Illumina, Inc, San Diego, CA). The post-PCR library was then used for exome capture using the Agilent SureSelect 50 Mb Exome Capture kit (Santa Clara, CA, USA). Exome enriched products were sequenced using an Illumina HiSeq 2000 (Illumina, Inc, San Diego, CA) by Centrillion Biosciences Inc. (Mountain View, CA, USA). One sample was sequenced per lane to obtain an average theoretical depth of 100X, using 2×75 bp sequencing.

### Bioinformatics

Reads passing Illumina chastity filter, were subjected to a quality filter step that removed low quality bases from the 3′ end, and retained pairs of reads if the trimmed reads for both members of the pair were 50 bp or longer. Paired reads that passed the quality filter, were mapped to the reference human genome sequence (hg19) with GSNAP (Genomic Short-read Nucleotide Alignment Program, version 2012-05-07). [21] Sequence calls for variants (single-nucleotide polymorphisms (SNPs), insertions and deletions (indels) were performed using the GATK (Broad’s Genome Analysis Toolkit, version 1.6-11-g3b2fab9). [22].

After variant detection, the program ANNOVAR (Annotate Variation, version 2012-03-08) was used to classify variants (e.g., exonic, intronic, synonymous, non-synonymous, splice variant, stop gain, stop loss, insertion, or deletion) and to cross-reference all the variants across various genetic variation databases. Included in ANNOVAR are databases to determine nonsynonymous & splice site variants (refGene.txt), variants in conserved genomic regions (phastConsElements46way.txt), variants in segmental duplications (genomicSuperDups.txt) and variants (hg19_ALL.sites.2012_02.txt). Additionally ANNOVAR cross referenced variants to the 1000 genomes and NHLBI Exome Sequening Project (ESP) databases; variants not reported in either database were considered ‘novel’ for filtering purposes. [23] Only non-synonymous changes (SNPs and in-dels), those that cause an alternate splice site, and/or an aberrant stop codon were considered for further analysis. For non-synonymous changes, all insertion and deletion variants were considered damaging, whereas SNP variants were cross-referenced to the dbNSFP (database for nonsynonymous SNPs’ functional predictions, version 2.0b2) to determine whether the changes to the protein structure would be considered tolerable or damaging using four algorithms (Sorting Intolerant From Tolerant (SIFT), PolyPhen-2 (Polymorphism Phenotyping v2 ), likelihood ratio test (LRT), MutationTaster). [24].

The final filtered list of variants for each affected family member was then intersected to find putative causal variants. All putative mutations were confirmed with custom designed Sanger Sequencing methods. Sanger sequencing (primers available upon request). was used to confirm putative mutations identified by the bioinformatics analysis in the tested subjects and in all other affected relatives. PCR assays for neighboring single-nucleotide polymorphisms (SNPs) flanking the putative mutation (rs1104859, rs2365652, rs2275860, rs3767546, and rs3729547) were designed and Sanger sequencing was used to determine common haplotypes in subjects (Table S1). The deep-sequencing dataset was deposited in the NIH Short Read Archive (SRA) with the accession number PRJNA202882.

## Results

### Family Phenotypes

The clinical features and the outcome of the two families with DCM, AD-FDC1 and AD-FDC27 are described in Table 1. The affected family members presented typical clinical features consistent with DCM, with left ventricular dilatation and systolic dysfunction, responding to heart failure medication including beta-blockers and ACE-inhibitors during the long-term follow-up (ranging from 6 to 25 years). The male to female ratio was 2.6 in spite of the autosomal dominant transmission, as previously observed in familial DCM. The age of onset was very variable ranging from 26 to 90 years, suggesting a significant variability in age-related penetrance. Patients presented symptoms of heart failure, conduction disease and ventricular arrhythmias, but no increase in serum CK levels was observed in any of them to suggest a skeletal muscle involvement. The AD-FDC1 family was followed-up clinically for over two decades but no causative mutation was found; over the course of the follow-up, two individuals in an additional generation (V:3 and V:4, Figure 1A) developed DCM phenotypes.

**Table 1:** Clinical features of key member of family AD-FDC1 and AD-FDC27.

Family and Pedigree ID	Sex	Age at enroll-ment	Follow-up (years)	Symptoms at enrollment	Arrhythmia	NYHA	ECG	LVEDD (cm)	FS%	LVEF%	Outcome
**AD-FDC1**											
III:3	M	94	4	DOE	PVC	2	Incomplete LBBB	N/A	N/A	N/A	CHF death
lll:6	F	82	11	None	PVC	1	Incomplete LBBB	6	32	62	NYHA 2–3, LVEDD 6.4 cm, LVEF 37%, CHF death
III:8	M	67	6	Shortness of breath	Frequent PVC	2	AF, LBBB	6.2	15	20	NYHA 3, CHF death
lV:2	M	69	15	DOE, orthopnea	AF	2–3	AF	6.3	13	35	NYHA 3
lV:4	M	46	7	Fatigue	NSVT	2	Normal	6	13	42	NYHA 1, LVEF 51%
lV:6	F	35	22	DOE, PND	NSVT	3	PVC, LAFB	7.1	6	15	NYHA 1, LVEF 46%
lV:7	M	33	25	Abnormal X-ray	NSVT	1	1st degreeAVB, LAFB	8.7	11	17	NYHA 1, LVEF 40%, NSVT
lV:13	M	52	14	DOE, palpitations	NSVT	2	ST changes	6.9	25	43	NYHA 3, LVEF 27%, CHF death
lV:14	M	45	20	DOE	Frequent PVC	1	PVC	7	21	41	NYHA 2, LVEF 47%, Frequent PVC
lV:16	M	42	7	None	None	1	Normal	6.4	17	40	NYHA 2, LVEF 36%
V:3	M	1	21	None	None	1	Normal	N/A	28	76	NYHA 1, LVEF 44%
V:4	F	4	21	None	None	1	Normal	4.1	29	68	NYHA 1,LV dilatation
**AD-FDC27**											
II:6	M	54	8	None	None	1	LAFB	6.4	23	54	SD
II:8	M	57	11	DOE	SSS, PM	1	PM	7.1	N/A	29	NYHA 2
II:11	M	35	18	DOE	None	1	Normal	6.9	17	16	NYHA 4, CHF death
III:7	M	8	15	None	None	1	Normal	4	30	60	Cardiac arrest
III:8	F	21	23	Syncope	N/A	1	Normal	4.7	N/A	48	Death (accident)
III:9	F	25	15	Palpitations, syncope	None	1	RBBB	3.8	37	Normal	DCM, LBBB

NYHA - New York Heart Association class; ECG - Electrocardiogram; LVEDD - left ventricular end-diastolic diameter; LVEF - left ventricular ejection fraction; FS - fractional shortening; DOE - Dyspnea on exertion, PND - Paroxysmal nocturnal dyspnea, DCM - Dilated cardiomyopathy, PVC - Premature ventricular contractions, LBBB - Left bundle branch block, CHF - Congestive heart failure, SD - Sudden death, NSVT - Non-sustained ventricular tachycardia, PM - Pace maker, RBBB - Right bundle branch block, AF - Atrial fibrillation, AVB - 1st degree atrio-ventricular block, LAFB - Left anterior fascicular block, SSS - sick sinus syndrome.

### Exome Sequencing

Three affected individuals from family AD-FDC1 and six affected individuals from family AD-FDC27 underwent exome sequencing (Figure 1) and bioinformatics filtering (Table 2). In family AD-FDC1, sequencing yielded a mean coverage of 40.02X, 40.68X and 44.72X per base across the whole exome, for samples lV:4 and lV:7 and lV:14 respectively. Percentage of on target reads were 67.07% (IV:7), 66.36% (IV:14) and 67.99% (IV:4). The percentage and number of reads mapped before and after filtering for each subject sequenced by NGS are provided in Table S2. Ten other members from the family were selected for genotyping of putative mutations by traditional Sanger-sequencing to confirm segregation of the variants. Of the 13 total tested subjects, twelve had been diagnosed with DCM at the time of enrollment.

**Table 2:** Bioinformatics filtering algorithm.

			Pedigree ID			
			IV:4	IV:7	IV:14	Mean
		**Total aligned variants**	284,085	257,691	278,351	273,376
		**Number of indels before filtering**	8803	6594	7036	
			↓	↓	↓	↓
**Decision**	**Analysis Tool**	**Filtration Steps**				
Keep	ANNOVAR	Nonsynonymous, nonsense, & splice site variants	11,287	11,883	11,803	11,658
			↓	↓	↓	↓
Keep	ANNOVAR	Located in conserved regions	5,198	4,984	4,935	5,039
			↓	↓	↓	↓
Exclude	ANNOVAR	Located in segmental duplications	4,531	4,072	4,073	4,225
			↓	↓	↓	↓
Keep	ANNOVAR	Novel variants	484	174	197	285
			↓	↓	↓	↓
Keep	dbNSFP	Predicted to be damaging	230	85	93	136

Shown are bioinformatics analysis tools used and individual and mean variant numbers at each step of filtering with resulting decrease in variant numbers at each decision step.

Exome sequencing followed by bioinformatics and segregation analysis of family AD-FDC1 was done to identify a list of potential mutations shared by all three affected individuals. The bioinformatics analysis focused on rare missense, nonsense, and splicing variants predicted to affect the expected protein product.

**Figure 2:**
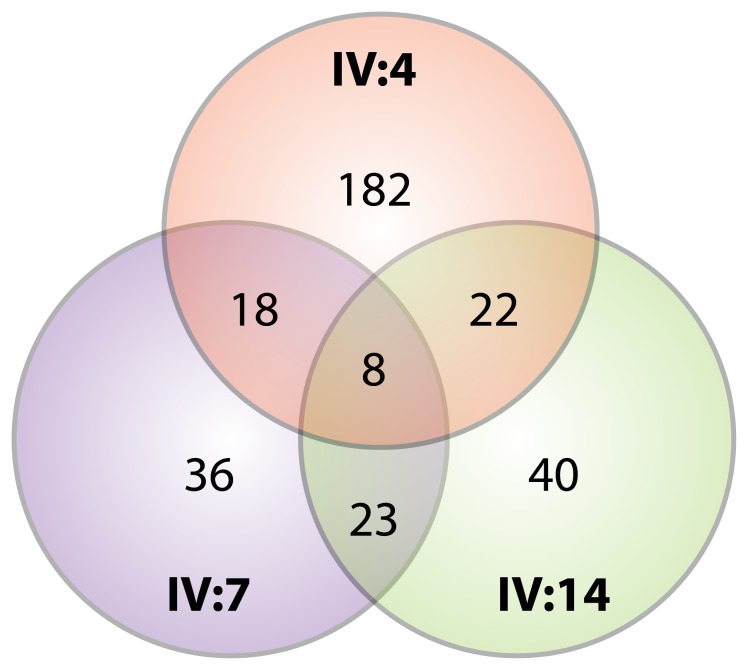
Venn diagram reflecting variant overlap between and among patients. A Venn diagram depicts the number of variants after bioinformatics filtering to identify nonsynonymous, nonsense and splice site variants that were located in conserved regions (by ANNOVAR), novel (absent in 1000 Genomes and NHLBI Exome Sequencing Project datasets), and were predicted to be damaging by in silico analyses. The numbers and overlap regions in the Venn diagram show the variants unique to or shared among three individuals (IV:4, IV:7, and IV:14) from family AD-FDC1.

Initially, two family members lV:7 and lV:14 were sequenced yielding 23 shared variants (Figure 2), from an average of 136 variants with damaging prediction per individual. Addition of a third affected relative, lV:4, further reduced the number of shared variants from an average of 21 for any two individuals to eight variants shared by all three affected relatives. Variants were next studied using Sanger sequencing and two variants were not re-confirmed by Sanger sequencing and represented false positives; the remaining six variants were then further studied by segregation analyses across all available affected family members (Table 3). Prediction scores from the five algorithms used by dbNSFP (SIFT, Polyphen 2 HDIV and HVAR, LRT, and MutationTaster) are presented in Table 3. Scores from all algorithms consistently predicted all variants to be damaging, except for UBLCP1, which was predicted to be possibly damaging by Polyphen2 HVAR.

**Table 3:** Resultant variants and dbNSFP scores after bioinformatic filtering in AD-FDC1.

Gene	Seg. within family	Chr.	Position	Exon	NM #	Variant	AminoAcid	SIFTscore	Polyphen 2HDIV score	Polyphen2HVAR score	LRTscore	MutationTaster score
*DUOX2*	no	15	45,386,870	33	14080	c. 4415 T>G	F1472C	0.01	1	0.998	0	0.999883
*DDIT4L*	no	4	101108877	3	145244	c. 539 delA	K180fs	n/a	n/a	n/a	n/a	n/a
*MAPKBP1*	no	15	42,105,214	8	14994	c. 734 C>T	A245V	0	1.0–0.933	0.975–0.448	0.000014	0.919339
*TNNT2*	yes	1	201,332,477	11	364	c. 517 C>T	R173W	0	1.0–1.0	0.994–0.992	0.000001	0.996697
*UBLCP1*	no	5	158,710,251	10	145049	c. 833 G>A	R278H	0.01	0.954	0.498	0	0.999844
*USP53*	no	4	120,169,945	6	19050	c. 280 C>G	P94A	0.11	1	0.999	0.000002	0.509941

Shown are the six genes containing exome-detected variants present in all three subjects tested. The Genebank NM number, chromosome and nucleotide position are shown along with the predicted consequence of each variant. Only the TNNT2 variant segregates with the disease phenotype in AD-FDC1. SIFT, Polyphen2, LRT, and MutationTaster scores derived from dbNSFP are presented. SIFT scores less than 0.05 are predicted to be damaging, otherwise they are predicted to be tolerated. Polyphen2_HDIV_scores range from 0 to 1. Scores in the range of 0.957 to 1 are predicted to be possibly damaging and those in the range of 0.453 to 0.956 are predicted to be benign. Polyphen2_HVAR_scores range from 0 to 1. Scores in the range of 0.909 to1 are predicted to be possibly damaging and scores in the range of 0.447 to 0.908 are predicted to be benign. Lower LRT p-values correspond to predictions that are more damaging. A MutationTaster value close to 1 indicates a high ‘security’ of the prediction.

### Segregation Analysis

**Figure 3:**
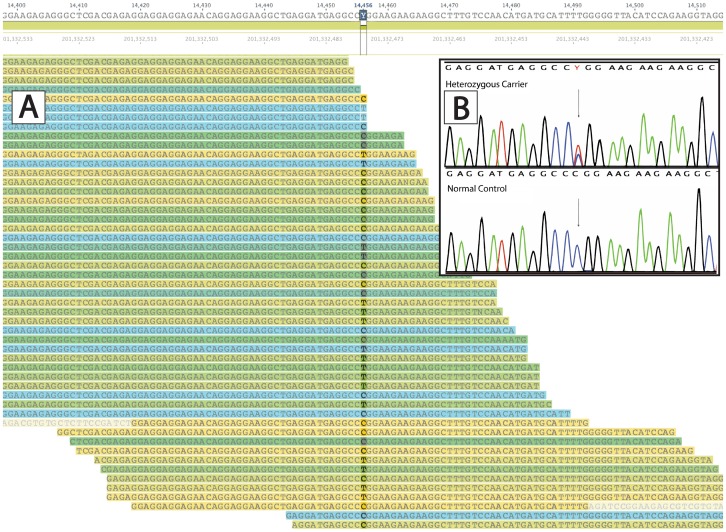
Visualization of NGS alignment and chromatogram from Sanger sequencing confirming the *TNNT2 Arg173Trp* variant. The alignment and Sanger sequencing profiles of the TNNT2 R173W variant are shown. A) C>T variant alignment reads of Arg173Trp variant B) (inset) Chromatogram of C>T variant of Arg173Trp variant from Sanger sequencing; arrow depicts the c.517T C>T (chr1∶201,332,477) position.

Only one variant was found to segregate appropriately across DCM-affected subjects, being shared by all affected and none unaffected individuals, *TNNT2*, c.517T C>T (chr1∶201,332,477; hereafter, Arg173Trp) (Figure 3a and 3b). Twelve affected individuals across the five-generation pedigree, including affected fourth-degree relatives carried the Arg173Trp variant, strongly supporting that this variant was responsible for the DCM phenotype in AD-FDC1. One relative who did not harbor the Arg173Trp variant (IV:5) had initially a ‘borderline’ phenotype (left ventricular ejection fraction of 45%) which did not progress in over 21 years of followup. [19] An analysis of 68 families in our registry detected the Arg173Trp variant co-segregating in one other large Italian family with DCM, AD-FDC27 (Figure 1B). To evaluate the possibility that the Arg173Trp variant could be a rare benign variant (minor allele frequency <1%) found in two separate families we completed exome sequencing in AD-FDC27 and excluded any other potential variant in other known or novel cardiomyopathy genes, further strengthening the Arg173Trp variant as the most probable pathogenic variant in this family. Although recurrent mutations are uncommon in DCM, in both families AD-FDC1 and AD-FDC27, known DCM causing genes had previously been evaluated using denaturing high-performance liquid chromatography (*LMNA, SGCD, DES, MYH6, MYH7, BANF1, EME1, LDB3, MYBPC3, SCN5A, TMPO* and *TTN*). Further, unbiased confirmation of the role of variant *TNNT2*, c.517T C>T was obtained by exome sequencing also in family AD-FDC27. Combining the segregation data across the two families yields a cumulative LOD score of 9.3 for Arg173Trp. Although the Arg173Trp variant would presumably be detected by current modern genetic testing DCM gene panels, the exome strategy and bioinformatics approach confirmed in an unbiased way the association of this recurrent variant with a DCM phenotype in two unrelated families.

### The Arg173Trp Variant was Located on Two Distinct Haplotypes

To determine whether AD-FDC1 and AD-FDC27, both families of Italian origin, were possibly related through the Arg173Trp variant on a shared haplotype, neighboring SNP typing was used to generate haplotypes. The four most common haplotypes from the HapMap dataset were detected across tested family members (Table 4). The Arg173Trp variant was found in the second-most (Haplotype 2) and most common haplotype (Haplotype 1) in families AD-FDC1 and AD-FDC27, respectively, arguing against a distant founder effect of the variant for these two families (Table S3).

**Table 4:** Hap Map project haplotypes and population frequencies of Utah residents with northern and western European ancestry (CEU).

Haplotype	rs1104859	rs2365652	rs2275860	rs3767546	rs3729547	Frequency
Chr. Position	201,331,554	201,331,664	201,333,703	201,333,961	201,334,382	–
1	C	T	G	T	T	0.637
2	A	G	G	T	C	0.196
3	A	G	A	T	C	0.088
4	C	G	G	A	T	0.078

The TNNT2, Arg173Trp variant (Chr1∶201,332,477) was present on separate haplotype, segregating with haplotypes #2 and #1 in families AD-FDC1 and AD-FDC27, respectively.

## Discussion

In this study, we demonstrated the utility of exome sequencing coupled with bioinformatic filtering and segregation analysis of the data to identify a rare, recurrent variant causative of adult-onset DCM in two families. In both our families, followed up for over 20 years, the affected family members showed a typical form of DCM: severe left ventricular dilatation and dysfunction, absence of skeletal muscle involvement and significant conduction disease. At the conclusion of the bioinformatics analysis, one variant, Arg173Trp remained consistent for causing the DCM phenotype in all tested families members. The Arg173Trp variation was not found in the 1,000 Genome Project cohort, as well as in the Framingham Heart Study and in Jackson Heart study cohorts (3,600 individuals) or in the NHLBI Exome Sequencing Project arguing against Arg173Trp being a rare benign variant. [25] PolyPhen-HCM, an algorithm that specifically predicts the effects of missense mutations in sarcomeric genes associated with hypertrophic cardiomyopathy (HCM), also scored Arg173Trip as pathogenic. [26] The Arg173Trp variant was present in one other DCM family in our registry and has been reported by others in DCM, [27] consistent with Arg173Trp being a recurrent *TNNT2* DCM mutation. The haplotype analysis of our two families indicates that the Arg173Trp variants most likely arose from separate mutation events on the two most common local haplotypes. A mutation at the same amino acid position (Arg173Gln) [28], [29] has been reported in other unrelated DCM families, suggesting a strong association of this residue with the disease, the existence of a recurrent mutation within the *TNNT2* gene, and that changes in this region of troponin T, proximal to one of the tropomyosin bindings sites, lead to DCM. A mutation at the preceding residue, Ala172Ser, was also reported by two groups in cardiomyopathy. [30], [31] Recently, Sun et al. generated induced pluripotent stem cells (iPS) from a DCM patient with the same Arg173Trp variant and demonstrated sarcomeric disorganization, depressed contractility, altered calcium ion regulation and increased susceptibility to inotropic stress, offering insight into the functional nature of this variant. [27] These data are in line with previous reports suggesting that *TNNT2* mutations leading to DCM cause decreased calcium sensitivity in the myofilaments and consequent decreased contractility. [32], [33] Interestingly, Sun et al. found that DCM iPS-derived cardiomyocytes were more susceptible to β-adrenergic stimulation (norepinephrine) while that their treatment with the β1-selective β-blocker metoprolol improved their disorganized sarcomeric pattern, recapitulating the clinical features observed in our DCM patients.

Although a powerful method, exome sequencing presents several challenges. In spite of multiple bioinformatics filtering steps, the results of exome sequencing often produce multiple variants that must be further adjudicated. In the case of autosomal recessive diseases, it is possible to filter variants by restricting the analysis to homozygous changes or to separate mutations within the same gene. Autosomal dominant conditions like DCM, where only singular heterozygous mutations are expected require a slightly different approach. Our data show the utility of leveraging distant affected relatives to narrow the list of candidate variants within a family. For example, when two individuals were sequenced the number of variants with damaging predictions was reduced from an average of 136 per individual down to an average of 21 shared between two individuals. This number dropped to eight variants when a third individual was added, highlighting the value of adding additional distant relatives. Clinical exome sequencing in small families or in single individuals might therefore pose challenges for confidently identifying the single causative DCM variant in many cases.

A limitation of exome sequencing is the bias towards coding regions, which do not represent the location of all causative mutations. This same limitation is present in current DCM-gene panels, projecting that both current and evolving exome approaches will continue to fall short of 100% sensitivity for the diagnosis of genetic forms of DCM. The challenges presented with potentially tens to hundreds of rare variants coming from exome sequencing should temper enthusiasm for using exome testing to replace current methods. However, in situations where distant affected relatives may be tested in parallel, exome sequencing may have an emerging clinical role. The development of multiple databases of exome and genome information is also improving the understanding of rare genetic variants and these data will progressively improve knowledge of rare but likely benign variants and will further argue for exome sequencing being a clinical diagnostic tool in DCM.

## Supporting Information

Table S1 **PCR primers for SNPs rs1104859, rs2365652, rs2275860, rs3767546, rs3729547.**
(DOCX)

Table S2 **Number of reads mapped, mean, standard error and percentage of reads mapped for each subject exome sequenced.** Shown are numbers of reads mapped before and after filtering, duplicate reads, percent yield and mean coverage. SD-standard deviation.
(DOCX)

Table S3 **Haplotypes of the affected member of family AD-FDC1 and AD-FDC27.** Haplotypes are numbered 1–4 by their increasing frequency in the population using the CEU dataset from the HapMap project (http://hapmap.ncbi.nlm.nih.gov/index.html.en). * refers to individuals with recombinant haplotypes (AD-FDC1: IV-12 had combined 1 and 2; AD-FDC27: Individual II-3 had combined haplotype 1 and 4; III-2 had combined haplotype 1 and 2).
(DOCX)
