# Supplementary material for: Whole Exome Sequencing Identifies a Troponin T Mutation Hot Spot in Familial Dilated Cardiomyopathy
Source: PLoS One. 2013 Oct 29;8(10):e78104. doi: 10.1371/journal.pone.0078104 (PMC3812167; doi:10.1371/journal.pone.0078104)
Supplement: Table S1 — PCR primers for SNPs rs1104859, rs2365652, rs2275860, rs3767546, rs3729547. (DOCX) [file pone.0078104.s001.docx]

**Table S1:** PCR primers for SNPs rs1104859, rs2365652, rs2275860, rs3767546, rs3729547.

| **SNP** | **Forward Primer** | **Reverse Primer** |
| --- | --- | --- |
| rs1104859 | 5`gtagggggctacaggcagag 3` | 5`caggagggccaggttcttat 3` |
| rs2365652 | 5`gtagggggctacaggcagag 3` | 5`caggagggccaggttcttat 3` |
| rs2275860 | 5`gggctgtggactgattgg 3` | 5`ggagggttccttccagagtc 3` |
| rs3767546 | 5`gggctgtggactgattgg 3` | 5`ggagggttccttccagagtc 3` |
| rs3729547 | 5`cagtccctgggtccagaat 3` | 5`atgttaggtgggcagactgg 3` |
