# Supplementary material for: Whole Exome Sequencing Identifies a Troponin T Mutation Hot Spot in Familial Dilated Cardiomyopathy
Source: PLoS One. 2013 Oct 29;8(10):e78104. doi: 10.1371/journal.pone.0078104 (PMC3812167; doi:10.1371/journal.pone.0078104)
Supplement: Table S2 — Number of reads mapped, mean, standard error and percentage of reads mapped for each subject exome sequenced. Shown are numbers of reads mapped before and after filtering, duplicate reads, percent yield and mean coverage. SD-standard deviation. (DOCX) [file pone.0078104.s002.docx]

**Table S2:** Number of reads mapped, mean, standard error and percentage of reads mapped for each subject exome sequenced

| **Sample ID** | **Before filtering** | **After filtering (%)** | **Properly mapped (%)** | **# of duplicate reads (%)** | **Mapped (no duplicates)** | **Final % Yielded** | **Exome Mean coverage (SD)** |
| --- | --- | --- | --- | --- | --- | --- | --- |
| **Family: ADFDC001** |  |  |  |  |  |  |  |
| IV:7 | 69,576,762 | 64,526,420 (92.74) | 57,679,380 (89.42) | 1,488,006 (97.42) | 56,191,374 | 80.76% | 25.62 (0.00470) |
| IV:14 | 78,190,580 | 72,057,434 (92.16) | 64,607,840 (89.70) | 1,836,562 (97.16) | 62,771,278 | 80.28% | 28.15 (0.00525) |
| IV:4 | 109,798,268 | 108,792,299 (99.08) | 90,304,840 (83.1) | 38,748,735 (57.09) | 51,556,105 | 46.96% | 21.83 (0.00180) |
| **Family: TSFDC027** |  |  |  |  |  |  |  |
| II:6 | 59,249,954 | 57,012,552 (96.2) | 48,679,208 (85.94) | 24,082,834 (50.53) | 24,596,374 | 41.51% | 23.10 (0.00257) |
| II:11 | 19,609,084 | 18,906,792 (96.42) | 16,480,332 (88.89) | 8,083,633 (50.95) | 8,396,699 | 42.82% | 7.97 (0.00084) |
| II:8 | 20,390,466 | 19,712,650 (96.68) | 17,083,964 (88.66) | 5,433,821 (68.19) | 11,650,143 | 57.14% | 11.23 (0.00119) |
| III:9 | 91,491,280 | 89,690,872 (98.03) | 79,277,108 (88.43) | 9,146,631 (88.46) | 70,130,477 | 76.65% | 65.69 (0.00633) |
| III:8 | 74,064,932 | 72,351,896 (97.69) | 63,705,464 (88.11) | 7,363,516 (88.44) | 56,341,948 | 76.07% | 49.07 (0.00463) |
| III:7 | 32,737,848 | 32,016,956 (97.80) | 27,667,136 (86.44) | 2,836,485 (89.75) | 24,830,651 | 75.85% | 22.15 (0.00229) |
