# Supplementary material for: Whole Exome Sequencing Identifies a Troponin T Mutation Hot Spot in Familial Dilated Cardiomyopathy
Source: PLoS One. 2013 Oct 29;8(10):e78104. doi: 10.1371/journal.pone.0078104 (PMC3812167; doi:10.1371/journal.pone.0078104)
Supplement: Table S3 — Haplotypes of the affected member of family AD-FDC1 and AD-FDC27. Haplotypes are numbered 1–4 by their increasing frequency in the population using the CEU dataset from the HapMap project (http://hapmap.ncbi.nlm.nih.gov/index.html.en). * refers to individuals with recombinant haplotypes (AD-FDC1: IV-12 had combined 1 and 2; AD-FDC27: Individual II-3 had combined haplotype 1 and 4; III-2 had combined haplotype 1 and 2). (DOCX) [file pone.0078104.s003.docx]

**Table S3.** Haplotypes of the affected member of family AD-FDC1 and AD-FDC27

| **Family and Pedigree ID** | **rs1104859 (A/C)** | **rs2365652 (G/T)** | **rs2275860 (G/A)** | **rs3767546 (T/A)** | **rs3729547 (C/T)** | **Haplotype** |
| --- | --- | --- | --- | --- | --- | --- |
| **AD-FDC1** |  |  |  |  |  |  |
| III-3 | A C | G G | G G | T A | C T | 2 4 |
| III-6 | A C | G G | G G | T A | C T | 2 4 |
| IV-2 | A C | G T | G G | T T | C T | 1 2 |
| IV-4 | A A | G G | G A | T T | C C | 3 2 |
| IV-5 | C A | T G | G G | T T | T C | 1 2 |
| IV-6 | A C | G G | G G | T A | C T | 2 4 |
| IV-7 | A A | G G | G G | T T | C C | 2 2 |
| IV-12* | C C | T G | G G | T A | C T | 12 4 |
| IV-13 | A C | G T | G G | T T | C T | 1 2 |
| IV-14 | A C | G T | G G | T T | C T | 1 2 |
| IV-15 | C A | T G | G G | T T | T C | 1 2 |
| IV-16 | A C | G T | G G | T T | C T | 1 2 |
| V-3 | A C | G T | G G | T T | C T | 2 1 |
| **AD-FDC27** |  |  |  |  |  |  |
| II-1 | C C | T G | GG | T A | T T | 1 4 |
| II-3* | C C | T T | GG | T A | T T | 1 14 |
| II-7 | C C | T T | GG | T T | T T | 1 1 |
| II-9 | C C | T T | GG | T T | T T | 1 1 |
| II-13 | C C | T T | GG | T T | T T | 1 1 |
| III-2* | C C | T G | GG | T T | T C | 1 12 |
| III-10 | C A | T G | G A | T T | T C | 1 3 |
| III-11 | C C | T T | GG | T T | T T | 1 1 |
| III-12 | C C | T T | GG | T T | T T | 1 1 |
